# Exploring Salivary Alpha-Amylase as a Biomarker in Periodontitis: A Comparative Analysis of Disease Stages and Clinical Correlations

**DOI:** 10.3390/cimb46110726

**Published:** 2024-10-30

**Authors:** Nada Tawfig Hashim, Sadiah Fathima, Nurain Mohammad Hisham, Pooja Shivappa, Michael V. Magaogao, Md Sofiqul Islam, Sara Faisal Ahmed, Rasha Babiker, Muhammed Mustahsen Rahman

**Affiliations:** 1 Periodontics Department, RAK College of Dental Sciences, RAK Medical & Health Sciences University, Ras-AlKhaimah P.O. Box 12973, United Arab Emirates; sadiah.19902012@rakmhsu.ac.ae (S.F.); nurain.19902017@rakmhsu.ac.ae (N.M.H.); mustahsin@rakmhsu.ac.ae (M.M.R.); 2 Translational Medical Research Centre, RAK Medical & Health Sciences University, Ras-AlKhaimah P.O. Box 11127, United Arab Emirates; pooja@rakmhsu.ac.ae (P.S.); michael@rakmhsu.ac.ae (M.V.M.); 3 Operative Department, RAK College of Dental Sciences, RAK Medical & Health Sciences University, Ras-AlKhaimah P.O. Box 12937, United Arab Emirates; sofiqul.islam@rakmhsu.ac.ae; 4 Department of Statistics, Faculty of Mathematical Sciences, University of Khartoum, Khartoum 13314, Sudan; sara.faisal.ahmed@gmail.com; 5 Physiology Department, RAK College of Medical Sciences, RAK Medical & Health Sciences University, Ras-AlKhaimah P.O. Box 11127, United Arab Emirates; rashababiker@rakmhsu.ac.ae

**Keywords:** periodontal disease, salivary alpha-amylase, biomarkers, periodontitis, saliva diagnostics, clinical attachment loss, probing depth

## Abstract

Periodontal disease, characterized by bacterial plaque accumulation and subsequent immune response, can lead to gingivitis and periodontitis if untreated. Salivary alpha-amylase (sAA) has emerged as a potential biomarker with implications in periodontal disease progression. Objectives: This study aimed to assess and compare salivary alpha-amylase levels in individuals with periodontitis and healthy controls and to investigate its relationship with clinical parameters of periodontal disease. Forty-five participants were categorized into periodontally healthy (n = 13), Stage I and II Periodontitis (n = 17), and Stage III and IV periodontitis (n = 15) groups. Saliva samples were collected and analyzed using ELISA kits. Statistical analyses included tests for normality, group comparisons, post hoc analysis, and correlation analysis. Significant differences in salivary alpha-amylase levels were observed among severity groups (*p* < 0.05), with higher levels in periodontitis patients than healthy controls. Spearman correlation revealed moderate positive associations between alpha-amylase levels and probing depth (PD) and clinical attachment loss (CAL). Elevated salivary alpha-amylase levels were found to be associated with more severe periodontal disease, suggesting its potential as a biomarker for periodontitis severity. These findings support the utility of salivary biomarkers in periodontal disease diagnosis and monitoring, although further validation and standardization are warranted for clinical application.

## 1. Introduction

Periodontal disease is a chronic inflammatory condition affecting the supporting structures of the teeth, including the gingiva, periodontal ligament, and alveolar bone. It is initiated by the accumulation of bacterial plaque along the gingival margin, which triggers an immune response, leading to tissue inflammation and destruction if left untreated. The disease manifests in various forms, from gingivitis, characterized by gingival inflammation, to periodontitis, which involves irreversible damage to the supporting tissues and potential tooth loss [1].

Periodontal pocket depth (PD) and clinical attachment loss are key indicators of periodontitis, and radiographic imaging confirms the diagnosis by showing periodontal bone loss. The percentage of Gram-negative bacteria in the subgingival plaque increases in response to the loss of adhesion and osteolysis [2,3]. The primary pathogenic microorganism in subgingival plaque, such as Porphyromonas gingivalis, a Gram-negative bacterium, interacts with immune cells, primarily macrophages, to trigger an inflammatory response. Additionally, tissue destruction is caused by proteolytic factors (e.g., TNF-α and IL-8) released by these microbes [4].

Salivary alpha-amylase (sAA) is an enzyme found in human saliva that plays a crucial role in the initial digestion of starches, catalyzing the hydrolysis of complex carbohydrates into simpler sugars, like maltose and glucose, and facilitating their absorption in the digestive tract. Beyond its role in carbohydrate metabolism, sAA has gained attention for its potential implications in various physiological and pathological processes, including periodontal disease [5,6,7]. Research suggests that sAA may modulate the oral microbiome and host immune response. For instance, sAA has been shown to influence bacterial adhesion and biofilm formation on tooth surfaces, potentially impacting the pathogenesis of periodontal diseases [8].

**Figure 1:**
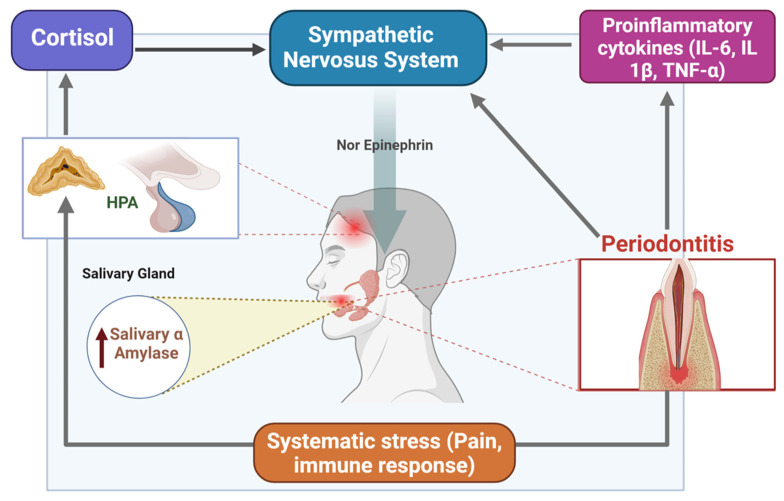
This figure illustrates the biological mechanisms through which salivary alpha-amylase (sAA) levels increase in response to periodontitis. Periodontitis triggers the release of pro-inflammatory cytokines (IL-6, IL-1β, and TNF-α), activating the sympathetic nervous system (SNS). This leads to the release of norepinephrine, which stimulates the salivary glands to produce more sAA. Systemic stress, caused by pain and immune response, further activates the hypothalamic–pituitary–adrenal (HPA) axis, leading to cortisol release, which also enhances SNS activity. The combined effect results in elevated sAA levels, linking periodontitis severity with stress and immune responses. Created by Biorender.com.

The increase in salivary amylase levels in periodontitis is primarily driven by the body’s inflammatory and stress responses, particularly through activating the sympathetic nervous system (SNS) [9]. Periodontitis, as a chronic inflammatory disease, triggers the release of pro-inflammatory cytokines such as interleukin-6 (IL-6), interleukin-1β (IL-1β), and tumor necrosis factor-alpha (TNF-α), which not only mediate tissue destruction in the periodontal tissues but also activate the SNS [2,3,4]. This SNS activation leads to the release of norepinephrine from sympathetic nerve endings, thus stimulating the beta-adrenergic receptors in the salivary glands, particularly in the acinar cells, resulting in increased secretion of sAA. Additionally, periodontitis induces systemic stress due to ongoing pain and immune responses. This systemic stress activates both the SNS and the hypothalamic–pituitary–adrenal (HPA) axis, further promoting the release of stress hormones such as cortisol, which enhances sympathetic activity and elevates sAA production [10,11,12] (Figure 1). As periodontitis severity increases, chronic inflammatory and stress responses intensify, leading to a proportional rise in sAA levels as part of the body’s effort to manage the inflammatory burden. Moreover, sAA levels have been associated with inflammatory mediators in gingival crevicular fluid, highlighting its involvement in local immune responses [13,14].

The American Academy of Periodontology (AAP) has introduced a staging and grading system to classify periodontal diseases based on severity, complexity, extent, and progression. Staging focuses on the severity and extent of the disease, while grading assesses the rate of progression and response to treatment [15]. This system provides a standardized framework for assessing and managing periodontal conditions.

Recently, saliva has emerged as a key diagnostic tool for various health conditions. As an oral fluid, saliva serves multiple functions, including maintenance of oral pH, taste, digestion, and antimicrobial activity [9,16]. Ongoing research investigates the potential of saliva components, such as mucin, electrolytes, and immunoglobulins, to serve as diagnostic markers for systemic and oral diseases [10,11,12]. Recent studies have highlighted the intricate interplay between periodontal disease and salivary biomarkers, including sAA [17]. Incorporating sAA measurements into diagnostic and therapeutic algorithms for periodontal disease management is promising for improving risk stratification, early detection, and personalized treatment approaches. By elucidating the role of sAA in the pathophysiology of periodontal disease, researchers aim to enhance our understanding of disease mechanisms and develop novel therapeutic strategies targeting salivary biomarkers [18].

The objective of this study was to investigate and compare salivary alpha-amylase levels and their relationship with clinical parameters in patients with periodontitis. This study tested the hypothesis that alpha-amylase levels in saliva may provide a grading system suitable for evaluating periodontitis severity.

## 2. Materials and Methods

A total of 45 individuals, including 13 periodontally healthy subjects, 17 with Stage I and II Periodontitis, and 15 with Stage III and IV periodontitis who were systemically healthy and non-smokers, participated in this study. The study participants were selected from those attending the Ras Al Khaimah College of Dental Sciences (RAKCODS) dental clinic from July 2023 to April 2024. The Ethics Committee of RAK Medical & Health Sciences University approved all protocols involving patients and healthy subjects with the code number (RAKMHSU-REC-222-2022/23-UG-D). The aim and flow of the study were explained to the participants, who signed a consent before they participated in the study. The inclusion criterion for the study group was the presence of established periodontitis according to the criteria by the periodontal disease classification system and Conditions 2018 [15]. Exclusion criteria included those with cardiovascular or intake of antibiotics or anti-inflammatory drugs within 6 months before the study, pregnant women, subjects with a history of smoking, or any form of tobacco and alcohol consumption. This study used a non-random technique, selecting samples by determining which subjects met the research criteria included in the study within a certain period.

### 2.1. Samples Collection and Periodontal Examination

Both adults with healthy gingiva and adults with periodontitis visiting RAKCODS were invited to participate in this study. Participants were asked about their medical and dental history and requested to complete a questionnaire. Subsequently, they underwent a clinical examination and periodontal charting of the oral cavity, followed by the collection of an unstimulated saliva sample. Three research team members then separately took measurements for the periodontal chart and diagnosed the staging and grading of the participant. The investigators engaged in inter- and intra-examiner calibration sessions before the commencement of the study. When a weighted κ value of >0.7 at a 95% confidence interval was obtained for each parameter, the readings were considered reliable. An OPG (Extra Oral X-Ray) was then assessed for accurate staging and grading of periodontitis. Participants with periodontitis were grouped into stages (I-IV) and grades (A–C) according to the new classification of periodontal disease (2017) [15]. Further categorization of the participants was performed based on the criteria proposed by the Centers for Disease Control and Prevention and the American Academy of Periodontology (CDC/AAP) was used [19,20].

Periodontal disease severity was assessed based on probing depth (PD) and clinical attachment loss (CAL) measurements. The participants were classified as having mild, moderate, or severe periodontitis using the thresholds established by the American Academy of Periodontology (AAP) [15]. PD and CAL were recorded for each tooth, and the most affected sites were used to determine the overall disease severity for each participant. This approach ensures that both the current status (PD) and the cumulative history of periodontal destruction (CAL) are captured in the severity classification.

Following the clinical examination and diagnosis of periodontitis, the participants were requested to provide an unstimulated saliva sample.

### 2.2. Saliva Sample Collection

For saliva collection, patients were instructed to rinse with tap water and spit; after that, they were seated in an upright position and asked to allow the saliva to collect at the floor of the mouth. They were then instructed to spit into the sample collection containers.

The saliva samples were stored at a temperature of −20 °C until processing.

In the laboratory, each saliva sample received was assigned a serial number and recorded. The samples were immediately clarified by centrifugation for 5 min at 1000× *g* (Heraeus Multifuge^®^ 4KR Centrifuge) (Thermo Fisher Scientific, Waltham, MA, USA). The supernatant was collected and aliquoted in 500 μL, using micropipettes, into clean microcap tubes (Microtube 2 mL, PP–Sarstedt, Germany). Two aliquots were made from each saliva sample and kept in an ultra-low-temperature freezer at −80 °C until processing (U725 Innova^®^ freezer, New Brunswick Scientific, Edison, WI, USA, last serviced by Biologic Solutions Limited (London, UK) in June 2015).

### 2.3. Measurement of Salivary Amylase

Biochemical analysis of amylase in saliva was performed using a salivary amylase test kit. The ELISA kits used for the study were purchased from ElabScience. Specifically, the ElabScience^®^ QuicKey ELISA^®^ Kit and the ElabScience^®^ QuicKey Pro™ were utilized. These kits employ the Sandwich-ELISA principle, which involves the capture of the target antigen between two layers of antibodies (capture and detection). The kits are designed for the quantitative detection of specific proteins in various sample types, including serum, plasma, and cell culture supernatants. The micro-ELISA plate provided in the kit comes pre-coated with an antibody specific to Human AMY1. Samples (or Standards) were added to the micro-ELISA plate wells and combined with the specific antibody. Then, a biotinylated detection antibody specific to Human AMY1 and Avidin-Horseradish Peroxidase (HRP) conjugate was added successively to each microplate well and incubated. Free components were washed away. The substrate solution was added to each well. Only those wells that contained Human AMY1, biotinylated detection antibody, and Avidin-HRP conjugate appeared blue. The concentration of Human AMY1 in the samples was calculated by comparing the OD of the samples to the standard curve. Glomax Explorer Micrplate Reader was the ELISA Reading Device.

### 2.4. Statistical Analysis

The statistical analysis of the study employed the following tests and methodologies:

- Normality test (Shapiro–Wilk test): This test was used to assess the normality of data distribution within each group.
- Homogeneity of variances (Levene’s test): Levene’s test was applied to evaluate the equality of variances across groups.
- Non-parametric test for group comparisons (Kruskal–Wallis Test): Due to non-normality in one group, the Kruskal–Wallis test was used to compare median differences across groups.
- Post hoc analysis (Mann–Whitney U test): Pairwise comparisons among groups were conducted using the Mann–Whitney U test, with Bonferroni correction applied to identify specific group differences.
- Correlation analysis (Spearman correlation coefficients): Spearman’s correlation coefficients were calculated to explore relationships between amylase levels, probing depth (PD), and clinical attachment loss (CAL).
- Mann–Whitney U test: This test was also used to compare amylase levels between healthy and periodontitis groups.

## 3. Results

### 3.1. Data Description

The current study included a total of 45 participants, classified according to the severity of their periodontal condition. Of these participants, 58% were male and 42% were female, with a mean age of 38.8 ± 8.8 years. The diagnoses ranged from healthy individuals to those with various stages and grades of periodontitis (Stage I to Stage IV; Grade A to Grade C) (Table 1).

The severity of periodontitis was categorized as mild, moderate, or severe, corresponding to the grade and stage of the disease as follows:

Mild periodontitis: This group included 10 subjects, characterized by clinical attachment loss (CAL) of 1–2 mm.

Moderate periodontitis: There were nine subjects in this group, with a CAL of 3–4 mm, representing a more advanced condition compared to the mild group but less severe than the severe periodontitis cases.

Severe periodontitis: This group, comprising 13 subjects, showed the highest level of periodontal destruction, with a CAL of more than 5 mm [20].

**Figure 2:**
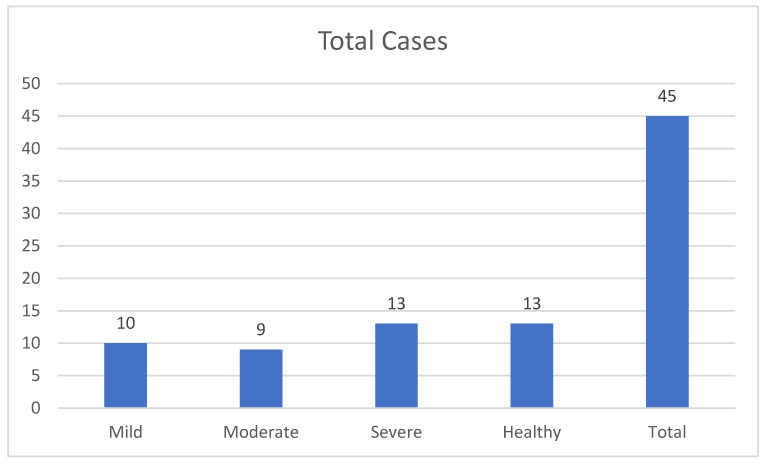
Shows the distribution of cases across different severity categories.

Healthy: The study also included 13 subjects with healthy periodontal tissues, defined by an intact periodontium without clinical attachment loss or bone loss [20] (Table 1 and Figure 2).

In the mild and moderate categories, PD values were 3.9 mm and 5.8 mm, respectively, while the severe group showed a PD of 7.33 mm. In contrast, the healthy group exhibited minimal PD and CAL values, with PD averaging 0.05 mm and CAL at 0.1 mm. Additionally, the average CAL was recorded at 1.8 mm in the mild group, 3.6 mm in the moderate group, and 5.5 mm in the severe group.

**Figure 3:**
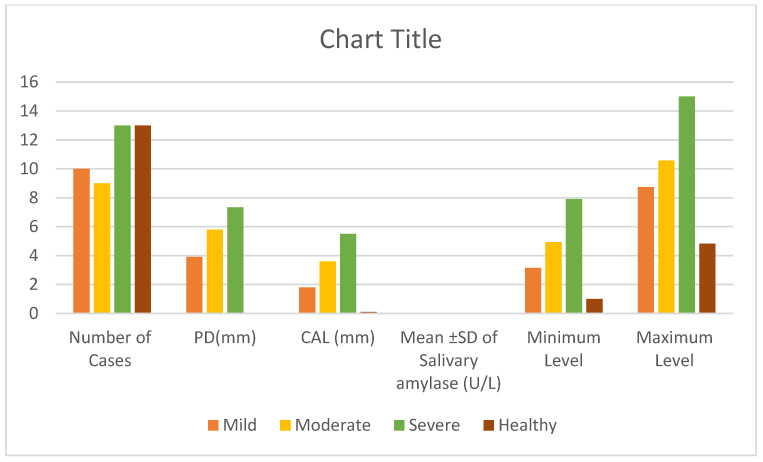
Summarizes Results for Salivary Alpha-Amylase Levels, Probing Depth (PD), and Clinical Attachment Loss (CAL) across Different Severity Levels of Periodontitis.

Salivary amylase levels were quantified, showing average concentrations of 4.70 U/L, 7.18 U/L, and 10.51 U/L in the mild, moderate, and severe periodontitis groups, respectively, compared to 3.3 U/L in the healthy group (Figure 3).

The study provides a detailed analysis of periodontitis severity, dividing participants into four groups: healthy, mild, moderate, and severe, with respective sample sizes of 13, 10, 9, and 13. Clinical measurements showed a progressive increase in pocket depth (PD) and clinical attachment loss (CAL) as the severity of periodontitis escalated. In the mild and moderate categories, the PDs were 3.9 mm and 5.8 mm, respectively, while in the severe category, the value reached 7.33 mm. In contrast, the healthy group showed minimal PD and CAL values of 0.05 mm and 0.1 mm. Additionally, the average attachment loss was 1.8 mm in the mild group, 3.6 mm in the moderate group, and 5.5 mm in the severe group. Salivary amylase levels were quantified, revealing average concentrations of 4.70 U/L, 7.18 U/L, and 10.51 U/L for mild, moderate, and severe periodontitis, respectively, and 3.3 U/L for the healthy group (Table 2).

### 3.2. Normality Test

The Shapiro–Wilk test was employed to assess the normality of data distributions within each group, and Levene’s test was used to evaluate the homogeneity of variances. For the mild severity group, the Shapiro–Wilk test statistic was 0.818, with a *p*-value of 0.024, indicating a departure from normality. In contrast, the moderate severity group had a test statistic of 0.869 and a *p*-value of 0.120, and the severe severity group had a test statistic of 0.888 with a *p*-value of 0.093, both suggesting that we cannot reject the null hypothesis of normality. Levene’s test for homogeneity of variances yielded a statistic of 1.41, with a *p*-value of 0.260, demonstrating no significant difference in variances among the groups, thus meeting the assumption of homogeneity. Given that the mild severity group did not pass the normality test, a non-parametric test, specifically the Kruskal–Wallis test, was conducted to compare median differences across the groups, as it does not assume a normal distribution of the data.

### 3.3. Comparison of Salivary Alpha-Amylase Levels Among the Groups

When a comparison in amylase levels among the three groups (mild, moderate, and severe) was performed, the Kruskal–Wallis test statistic was 22.12, with a *p*-value of approximately 0.0000157, indicating that there are statistically significant differences in the median amylase levels across the different groups (Table 3).

Given this significant result, the post hoc test was performed to identify which specific pairs of severity groups differ significantly. The Mann–Whitney U test was used for pairwise comparisons, with a correction for multiple testing (Table 4).

### 3.4. Post Hoc Analysis Results (Pairwise Mann–Whitney U Test with Bonferroni)

The results suggest significant differences in amylase levels among all pairwise comparisons of the groups, reinforcing the hypothesis that amylase could serve as a biomarker for the severity of periodontitis.

The data clearly show an upward trend in the mean amylase levels as the severity of periodontitis increases, and this observation could indicate that higher amylase levels are associated with more severe periodontal disease.

### 3.5. Correlation Analysis

**Figure 4:**
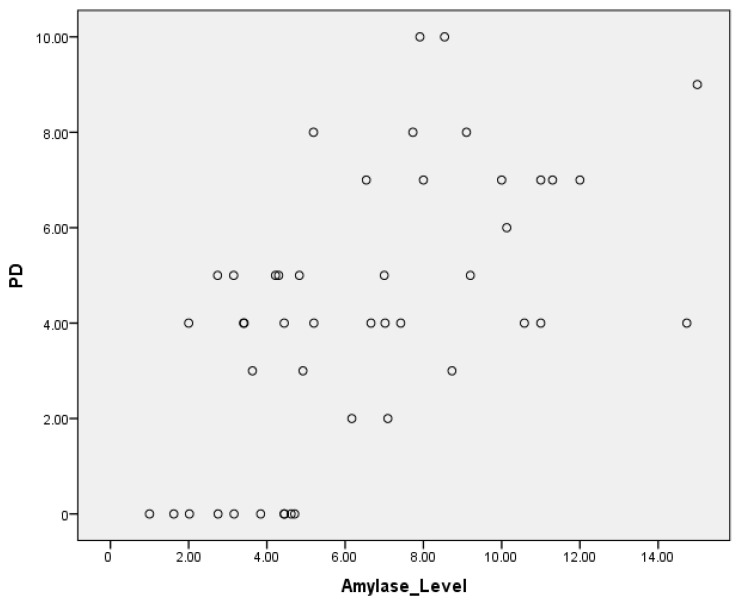
A scatter plot showing the correlation between salivary alpha-amylase levels and probing depth in periodontitis patients. A moderate positive correlation is revealed between the salivary alpha-amylase levels and PD.

**Figure 5:**
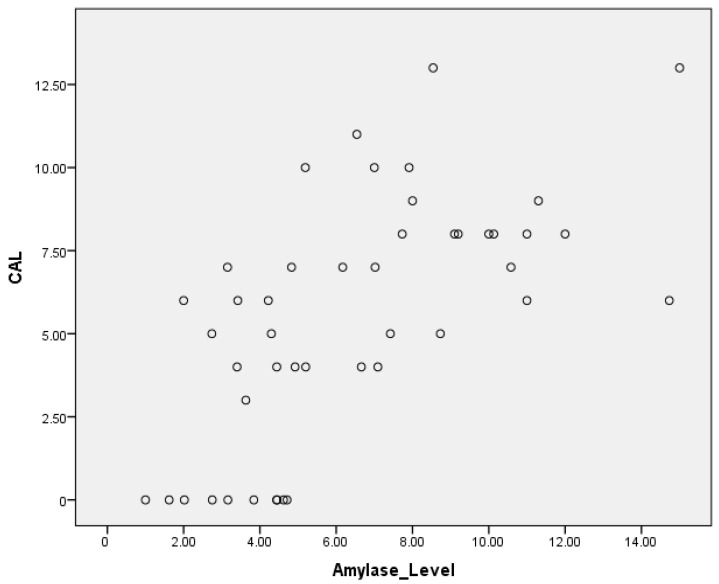
A scatter plot showing the correlation between salivary alpha-amylase levels and CAL in periodontitis patients. A moderate positive correlation is revealed between the salivary alpha-amylase levels and CAL.

The Spearman correlation coefficients were calculated to explore the relationships between amylase levels, probing depth (PD), and clinical attachment loss (CAL). The correlation between amylase levels and PD was found to be (r = 0.6), indicating a moderate positive correlation, suggesting that higher amylase levels tend to be associated with increased probing depth (Table 5). Similarly, the correlation between amylase levels and CAL was (r = 0.7), demonstrating a moderate positive correlation and indicating a significant association between higher amylase levels and greater clinical attachment loss (Table 6). These findings suggest that as both the stage and grade of diagnosis increase, there is generally an increase in the mean U/L values, and this increase may indicate a correlation between U/L values and the severity of the condition being diagnosed (Figure 4 and Figure 5).

### 3.6. Comparison of Salivary Alpha-Amylase Levels Among Periodontitis and Healthy Groups

When the analysis was performed to compare the level of amylase in both the healthy and periodontitis groups, it was found that the healthy group had a mean amylase level of 3.33, with a standard deviation of 1.30, while the periodontitis group had a mean amylase level of 7.82, with a standard deviation of 3.00. The Mann–Whitney U test results show a significant difference in amylase levels between individuals with healthy periodontal status and those with periodontitis. In the healthy group, the mean rank is 25.615, with a sum of ranks of 334 and a U statistic of 379. The *p*-value is reported as <0.01 (Table 7).

## 4. Discussion

### Comparison of Salivary Alpha-Amylase Levels as Biomarkers Among Different Groups of Periodontitis

This study categorizes periodontitis into mild, moderate, and severe based on clinical attachment loss and compares these with a healthy control group.

Our findings indicate that salivary alpha-amylase (SAA) levels are significantly elevated in moderate and severe periodontitis compared to healthy individuals. However, SAA levels in mild periodontitis do not significantly differ from healthy controls, suggesting that SAA alone may not be sufficient to detect early disease stages.

The findings that amylase levels correlate with the severity of periodontitis support the hypothesis that salivary biomarkers can be effective in diagnosing and monitoring periodontal disease [21].

The significant differences in amylase levels could suggest amylase’s potential as a biomarker for monitoring disease severity or progression. This aligns with the 2018 classification’s focus on disease progression and the need for markers that can predict disease trajectory or treatment response.

Several studies in the literature have investigated the relationship between amylase levels and periodontal disease. One study found significant differences in salivary alpha-amylase levels between subjects with gingivitis and those with periodontitis, suggesting that increased amylase levels could be associated with the progression and severity of periodontal disease [12,21].

Other studies compared the salivary alpha-amylase levels in chronic periodontitis patients to healthy controls. It was observed that salivary alpha-amylase levels were significantly higher in the chronic periodontitis group, while serum alpha-amylase levels remained consistent across both groups. This supports the potential use of salivary alpha-amylase as a biomarker for periodontal disease [22,23].

These findings align with the notion that biochemical markers in saliva, such as alpha-amylase, could provide insights into the systemic impact of periodontal diseases and offer a non-invasive method for monitoring disease progression and severity [24].

Research suggests that as periodontitis progresses from earlier stages (I and II) to more advanced stages (III and IV), the inflammatory response intensifies. This can stimulate the sympathetic nervous system, potentially leading to the increased secretion of alpha-amylase. The significant differences we found among stages may reflect this escalation in biological stress and inflammatory response [25].

These findings agree with the other studies that demonstrate increased salivary amylase in periodontitis patients, reinforcing the idea that salivary enzymes are reflective of the inflammatory state and, thus, can serve as reliable biomarkers for periodontal disease severity [12,26].

However, not all studies align perfectly with these results. For instance, a study by Parlak et al. found that while certain salivary proteins were elevated in periodontitis patients, amylase levels did not significantly differ across various stages of the disease [27].

This may be because the activity of amylase might be influenced by other factors, such as hydration status and flow rate of saliva, which were not controlled in their study.

Another contrasting finding comes from a systematic review by Kinney et al., which was inconclusive about the role of amylase, suggesting that while some studies show elevated amylase levels with increased periodontal inflammation, others do not see a clear pattern, possibly due to methodological differences and the heterogeneity of study populations [28].

The findings from the present study contribute to the ongoing debate about the utility of salivary biomarkers in periodontology. While there is supportive evidence that salivary amylase levels increase with the severity of periodontitis, contrasting findings suggest that not all studies have been able to replicate these results consistently. These discrepancies could be attributed to differences in sample sizes, the demographic characteristics of subjects, variations in disease definition and classification, and the sensitivity of the assays used [29,30].

Clinical attachment loss (CAL): This study defines the severity of periodontitis based on CAL, which is a critical measure used in the new classification to determine the stage of periodontal disease [15].

A Pearson correlation analysis between Amylase Level U/L and both PD and CAL was performed, and the results revealed moderate positive correlations between Amylase Level U/L and both PD and CAL.

The scatter plots illustrating salivary alpha-amylase (SAA) levels against probing depth (PD) and clinical attachment loss (CAL) reveal that high SAA values (>14 U/L) are present across a spectrum of disease severities, including both mild and severe cases. This suggests that individual variability in SAA levels may be influenced by factors beyond periodontal inflammation, such as systemic stress or genetic predisposition. While a moderate positive correlation was observed between SAA and both PD and CAL, these findings indicate that SAA alone may not be sufficient to accurately stratify disease severity in all individuals. Future studies should investigate additional salivary biomarkers and clinical parameters to provide a more comprehensive assessment of periodontal disease progression. Additionally, the scatter plot of clinical attachment loss (CAL) against salivary alpha-amylase (SAA) levels shows that individuals with severe periodontitis (CAL >5 mm) exhibit a wide range of SAA levels, from as low as 2 U/L to as high as 14 U/L. This variability suggests that while SAA is moderately correlated with CAL at a group level, individual SAA responses may be influenced by additional factors, such as systemic inflammation, stress, or genetic predisposition. The observed spread in SAA values within the severe disease category raises questions about the consistency of this relationship and suggests that the correlation may not be linear across all participants. Future studies should consider using multiple regression models to account for potential confounding factors and better understand the factors contributing to variability in SAA levels in advanced periodontitis.

The results of this study indicate that while salivary alpha-amylase (SAA) levels are significantly correlated with clinical parameters of periodontitis, the overlap in values across disease stages suggests that SAA alone is not sufficient to accurately determine disease progression. The moderate correlation observed between SAA and probing depth (PD) and clinical attachment loss (CAL) highlights the potential of SAA as part of a broader panel of biomarkers.

The Mann–Whitney U test was used to determine if there is a significant difference in amylase levels between individuals with healthy periodontal status and those with periodontitis and the result of the test revealed that there is a significant difference in amylase levels between individuals with healthy periodontal status and those with periodontitis.

This significant difference suggests that there may be a relationship between periodontal status and salivary amylase levels. Periodontitis is characterized by inflammation and destruction of the supporting structures of the teeth, leading to increased levels of inflammatory mediators in the gingival crevicular fluid and saliva [31].

Previous research has shown alterations in salivary composition, including amylase levels, in individuals with periodontal diseases compared to healthy controls [27,29].

Higher levels of salivary amylase in individuals with periodontitis could be attributed to increased salivary flow rates due to inflammation and immune responses in the oral cavity [31].

However, further research is needed to elucidate the underlying mechanisms and potential clinical implications of these findings.

Salivary alpha-amylase has not only been implicated in inflammatory and stress responses but also in pain, as highlighted in the recent literature. Research has shown that sAA levels rise in response to various forms of physical and emotional stress, including acute pain. In the study by Surin et al., elevated sAA levels were reported in patients undergoing mandibular third molar surgery, correlating significantly with their postoperative pain intensity. This finding emphasizes the role of sAA as a potential biomarker for pain, indicating that sAA secretion may be part of the body’s sympathetic response to surgical trauma and pain [32]. Similarly, Teja et al. reported that patients undergoing emergency endodontic treatment exhibited elevated sAA levels in correlation with the intensity of dental pain. This reinforces the concept that sAA could serve as a reliable marker of the physiological stress and pain experienced during dental emergencies [33]. Given the emerging evidence, it is plausible that in patients with periodontal disease, elevated sAA levels could also reflect an underlying response to pain. Periodontal disease often causes discomfort and inflammation, both of which may stimulate the sympathetic nervous system, further elevating sAA levels. Therefore, in addition to its role in reflecting inflammation and disease progression, the elevated sAA levels observed in our study may also be linked to the pain experienced by patients with more advanced stages of periodontitis. Future research should aim to investigate the potential dual role of sAA in both inflammation and pain management in periodontal diseases, which may enhance its utility as a comprehensive biomarker.

## 5. Conclusions

The significant correlations between salivary alpha-amylase levels and clinical markers of periodontal disease severity observed in this study suggest that sAA may have potential as a non-invasive biomarker for assessing periodontal disease severity. However, given the limitations of this study, further validation through longitudinal research and larger, more diverse populations is required before sAA can be confidently used as a clinical tool in periodontal diagnostics.

## 6. Limitation

While the study has provided meaningful insights into the role of salivary alpha-amylase (sAA) as a biomarker for periodontal disease, several limitations should be acknowledged:

- Sample size: The relatively small sample size of 45 participants, although sufficient for preliminary analysis, limits the generalizability of the findings. Future studies with larger cohorts are needed to validate the results across more diverse populations.
- The sample size for this study was not based on a formal power calculation, and as such, the study may not have been adequately powered to detect small or medium effect sizes. While significant correlations between salivary alpha-amylase (sAA) levels and periodontal disease severity were observed, these findings should be interpreted with caution due to the relatively small sample size. Future studies with larger sample sizes and formal power calculations are necessary to validate these results and ensure the robustness of the findings
- Cross-sectional design: This study employed a cross-sectional design, which provides a snapshot of the relationship between sAA levels and periodontal disease severity. However, it does not allow for the determination of causality. Longitudinal studies would be beneficial in understanding the dynamics of sAA levels over the course of disease progression and treatment.
- Influence of confounding factors: Factors such as diet, hydration status, and psychological stress can influence salivary alpha-amylase levels. While we attempted to control for some variables (e.g., excluding smokers and systemic diseases), we could not account for all potential confounders. Further studies should consider a more comprehensive assessment of these factors.
- Future research should incorporate validated stress and anxiety scales, such as the Perceived Stress Scale (PSS) or dental anxiety questionnaires, alongside salivary cortisol measurements, to better assess and control for the influence of stress on sAA levels.
- Lack of pain assessment: The study did not directly assess pain intensity in relation to sAA levels, despite emerging evidence suggesting that sAA is correlated with pain. Including pain assessment as a variable in future research could provide additional insights into the dual role of sAA in inflammation and pain.

## Figures and Tables

**Table 1:** Distribution of cases across different diagnoses and severity levels, providing a clear overview of the data.

Diagnosis	Mild	Moderate	Severe
Stage IV, Grade C	0	0	7
Stage III, Grade B	0	2	0
Stage III, Grade C	0	0	6
Stage I, Grade A	5	0	0
Stage II, Grade A	5	0	0
Stage II, Grade B	0	7	0

**Table 2:** Summarizes results for salivary alpha-amylase levels, probing depth (PD), and clinical attachment loss (CAL) across different severity levels of periodontitis.

Severity of Periodontitis	Number of Cases	PD (mm)	CAL (mm)	Mean ± SD of Salivary Amylase (U/L)	Minimum Level	Maximum Level
					(U/L)	(U/L)
Mild	10	3.9	1.8	4.70 ± 1.74	3.15	8.73
Moderate	9	5.8	3.6	7.18 ± 1.52	4.92	10.58
Severe	13	7.33	5.5	10.51 ± 2.33	7.91	15
Healthy	13	0.05	0.1	3.3 ± 1.3	1	4.83

**Table 3:** The Kruskal–Wallis test and the median salivary alpha-amylase levels for each group. Statistically significant differences between groups, as well as the median values of salivary alpha-amylase levels for mild, moderate, and severe cases of periodontitis.

Test Statistic	*p*-Value	Mild Median	Moderate Median	Severe Median
22.12417	0.001	4.26	7.02	10.13

**Table 4:** Displays the U statistic and the corresponding Bonferroni-corrected *p*-values for each pairwise comparison. All comparisons show significant differences, confirming that salivary alpha-amylase level differences among these groups are statistically significant.

Comparison	U Statistic	Corrected *p*-Value	Significant Difference
Mild vs. severe	3	0.00041	Yes
Mild vs. moderate	11	0.0187	Yes
Severe vs. moderate	110	0.00197	Yes

**Table 5:** Spearman’s rho correlation coefficients between salivary alpha-amylase levels and probing depth (PD). ** indicates significance at the *p* < 0.01 level.

	Correlations		
			PD
Spearman’s rho	Amylase_Level	Correlation Coefficient	0.577 **
		Sig. (2-tailed)	0
		n	45

**Table 6:** Spearman’s rho correlation coefficients between salivary alpha-amylase levels and clinical attachment loss (CAL). ** indicates significance at the *p* < 0.01 level.

	Correlations		
			CAL
Spearman’s rho	Amylase_Level	Correlation Coefficient	0.665 **
		Sig. (2-tailed)	0
		n	45

**Table 7:** Shows the mean rank, the sum of ranks, U statistic, and *p*-value for the Mann–Whitney U test comparing salivary alpha-amylase levels between individuals with healthy periodontal status and those with periodontitis.

Group	Mean Rank	Sum of Ranks	U Statistic	*p*-Value
Healthy	25.615	334	379	
Periodontitis	41.5	541	0.005	

## Data Availability

Data is contained within the article.

## References

[1] Könönen E., Gursoy M., Gursoy U.K. (2019). Periodontitis: A Multifaceted Disease of Tooth-Supporting Tissues. J. Clin. Med..

[2] Page R.C., Eke P.I. (2007). Case definitions for use in population-based surveillance of periodontitis. J. Periodontol..

[3] Hashim N.T., Linden G.J., Winning L., Ibrahim M.E., Gismalla B.G., Lundy F.T., El Karim I.A. (2017). Putative periodontal pathogens in the subgingival plaque of Sudanese subjects with aggressive periodontitis. Arch. Oral. Biol..

[4] Ertugrul A.S., Sahin H., Dikilitas A., Alpaslan N., Bozoglan A. (2013). Comparison of CCL28, interleukin-8, interleukin-1β and tumor necrosis factor-alpha in subjects with gingivitis, chronic periodontitis and generalized aggressive periodontitis. J. Periodontal Res..

[5] Caloian C.S., Șurlin P., Ciurea A., Pop D., Caloian B., Leucuța D.C., Țigu A.B., Rasperini G., Micu I.C., Stanomir A. (2024). Exploring Periodontal Conditions, Salivary Markers, and Systemic Inflammation in Patients with Cardiovascular Diseases. Biomedicines.

[6] Lima D.P., Diniz D.G., Moimaz S.A.S., Sumida D.H., Okamoto A.C. (2010). Saliva: Reflection of the body. Int. J. Infect. Dis..

[7] Nater U.M., Rohleder N. (2009). Salivary alpha-amylase as a non-invasive biomarker for the sympathetic nervous system: Current state of research. Psychoneuroendocrinology.

[8] Hojo K., Nagaoka S., Ohshima T., Maeda N. (2014). Bacterial interactions in dental biofilm development. J. Dent. Res..

[9] Ali N., Nater U.M. (2020). Salivary Alpha-Amylase as a Biomarker of Stress in Behavioral Medicine. Int. J. Behav. Med..

[10] Padmanabhan V., Islam M.S., Habib M., Abdulaziz Z., Goud M., Chaitanya N.C., Haridas S., Rahman M.M. (2023). Association between Salivary Cortisol Levels, Dental Anxiety, and Dental Caries in Children: A Cross-Sectional Study. Dent. J..

[11] Warren K.R., Postolache T.T., Groer M.E., Pinjari O., Kelly D.L., Reynolds M.A. (2014). Role of chronic stress and depression in periodontal diseases. Periodontol 2000.

[12] Sánchez G.A., Miozza V., Delgado A., Busch L. (2011). Determination of salivary levels of mucin and amylase in chronic periodontitis patients. J. Periodontal Res..

[13] Fábián T.K., Hermann P., Beck A., Frejérdy P., Fábián G. (2012). Salivary defense proteins: Their network and role in innate and acquired oral immunity. Int. J. Mol. Sci..

[14] Sari E., Bakkal M., Güven B. (2016). Relationship between salivary and gingival crevicular fluid alpha-amylase activity and glycemic control in type 2 diabetes mellitus patients. J. Periodontol..

[15] Tonetti M.S., Greenwell H., Kornman K.S. (2018). Staging and grading of periodontitis: Framework and proposal of a new classification and case definition. J. Periodontol..

[16] Locker D. (2007). Subjective reports of oral dryness in an older adult population. Community Dent. Oral. Epidemiol..

[17] Nassar M., Hiraishi N., Islam M.S., Otsuki M., Tagami J. (2014). Age-related changes in salivary biomarkers. J. Dent. Sci..

[18] Kikuchi T., Hayashi J.I., Mitani A. (2022). Next-Generation Examination, Diagnosis, and Personalized Medicine in Periodontal Disease. J. Pers. Med..

[19] Krishna A., Vadakkekuttical R.J., Radhakrishnan C., Parambath F.C. (2021). Correlation of Periodontal Inflamed Surface Area with Glycemic Status in Controlled and Uncontrolled Type 2 Diabetes Mellitus. World J. Clin. Cases.

[20] Eke P.I., Page R.C., Wei L., Thornton-Evans G., Genco R.J. (2012). Update of the case definitions for population-based surveillance of periodontitis. J. Periodontol..

[21] Carolina D.N., Rusyanti Y., Susanto A. (2017). Comparison of salivary alpha-amylase levels in gingivitis and periodontitis. Dent. J..

[22] Neha P.T., Maya M., R Purushottam S.P. (2018). Salivary Amylase as a Biomarker in Health and Periodontal Diseases. Int. J. Contemp. Med. Res..

[23] Allah F.A.A.A., Ahmed H.A.E. (2023). Evaluation of salivary alpha amylase activity in smokers with periodontitis, Khartoum state, 2023. Eur. J. Clin. Exp. Med..

[24] Hungund S.A., Desai V.B., Shah M., Shekar M.K., Deka A., Sarmah S. (2013). Efficacy of nonsurgical periodontal therapy affecting salivary biomarkers in non-diabetic and type 2 diabetic periodontitis patients. J. Oral. Biol. Craniofacial Res..

[25] Develioglu H., Korkmaz S., Dundar S., Schlagenhauf U. (2020). Investigation of the levels of different salivary stress markers in chronic periodontitis patients. J. Oral. Biol. Craniofacial Res..

[26] Parlak M.H., Buber E., Gur A.T., Karabulut E., Akalin F.A. (2023). Statherin and alpha-amylase levels in saliva from patients with gingivitis and periodontitis. Arch. Oral. Biol..

[27] Kinney J.S., Ramseier C.A., Giannobile W.V. (2017). Oral fluid-based biomarkers of alveolar bone loss in periodontitis. Ann. N. Y. Acad. Sci..

[28] Sánchez G.A., Miozza V.A., Delgado A., Busch L. (2013). Relationship between salivary mucin or amylase and the periodontal status. Oral. Dis..

[29] Acquier A.B., Pita A.K., Busch L., Sánchez G.A. (2015). Comparison of salivary levels of mucin and amylase and their relation with clinical parameters obtained from patients with aggressive and chronic periodontal disease. J. Appl. Oral. Sci..

[30] Anwar M., Alam B.F., Ali S., Tariq S.F., Aali K., Abrar E., Alotaibi D.H., Alsinaidi A.A., Alrahlah A., Vohra F. (2022). Evaluation of Salivary Mucin, Amylase, Protein Profile, and Periodontal Parameters among Hypertensive and Diabetic Patients. Appl. Sciences..

[31] Kejriwal S., Bhandary R., Thomas B., Kumari S. (2014). Estimation of levels of salivary mucin, amylase and total protein in gingivitis and chronic periodontitis patients. J. Clin. Diagn. Res..

[32] Surin W., Chatiketu P., Hutachok N., Srichairatanakool S., Chatupos V. (2022). Pain intensity and salivary α-amylase activity in patients following mandibular third molar surgery. Clin. Exp. Dent. Res..

[33] Teja K.V., Ramesh S., Janani K., Srivastava K.C., Shrivastava D., Natoli V., Di Blasio M., Cicciù M., Minervini G. (2023). Clinical correlation of salivary alpha-amylase levels with pain intensity in patients undergoing emergency endodontic treatment. BMC Oral. Health.

